# Methane, Carbon Dioxide and Nitrous Oxide Fluxes in Soil Profile under a Winter Wheat-Summer Maize Rotation in the North China Plain

**DOI:** 10.1371/journal.pone.0098445

**Published:** 2014-06-03

**Authors:** Yuying Wang, Chunsheng Hu, Hua Ming, Oene Oenema, Douglas A. Schaefer, Wenxu Dong, Yuming Zhang, Xiaoxin Li

**Affiliations:** 1 Key Laboratory of Agricultural Water Resources, Center for Agricultural Resources Research, Institute of Genetics and Developmental Biology, Chinese Academy of Sciences, Shijiazhuang, Hebei, China; 2 Department of Soil Quality, Wageningen University, Alterra, Wageningen, The Netherlands; 3 Key Lab of Tropical Forest Ecology, Xishuangbanna Tropical Botanical Garden, Chinese Academy of Sciences, Menglun, Yunnan, China; Tennessee State University, United States of America

## Abstract

The production and consumption of the greenhouse gases (GHGs) methane (CH_4_), carbon dioxide (CO_2_) and nitrous oxide (N_2_O) in soil profile are poorly understood. This work sought to quantify the GHG production and consumption at seven depths (0–30, 30–60, 60–90, 90–150, 150–200, 200–250 and 250–300 cm) in a long-term field experiment with a winter wheat-summer maize rotation system, and four N application rates (0; 200; 400 and 600 kg N ha^−1^ year^−1^) in the North China Plain.

The gas samples were taken twice a week and analyzed by gas chromatography. GHG production and consumption in soil layers were inferred using Fick’s law. Results showed nitrogen application significantly increased N_2_O fluxes in soil down to 90 cm but did not affect CH_4_ and CO_2_ fluxes. Soil moisture played an important role in soil profile GHG fluxes; both CH_4_ consumption and CO_2_ fluxes in and from soil tended to decrease with increasing soil water filled pore space (WFPS). The top 0–60 cm of soil was a sink of atmospheric CH_4_, and a source of both CO_2_ and N_2_O, more than 90% of the annual cumulative GHG fluxes originated at depths shallower than 90 cm; the subsoil (>90 cm) was not a major source or sink of GHG, rather it acted as a ‘reservoir’. This study provides quantitative evidence for the production and consumption of CH_4_, CO_2_ and N_2_O in the soil profile.

## Introduction

Atmospheric concentrations of carbon dioxide (CO_2_), methane (CH_4_) and nitrous oxide (N_2_O) have increased considerably since the industrial revolution, and are still increasing annually by about 0.5%, 1.1% and 0.3%, respectively [1]. Worldwide concerns about the increased greenhouse gases (GHGs) concentrations in the atmosphere and its effects on our future environment require a better understanding of the cause of these emissions [2]. Agricultural lands occupy 37% of the earth’s land surface; about 13.5% of global anthropogenic GHG was emitted from agricultural production [1]. It was estimated that 84% of N_2_O and 52% of CH_4_ emitted from agriculture activities [3]. In China, agriculture tends to produce more emissions than the global average over the last 30 years due to increased chemical and manure N inputs. Gaining a better understanding of GHG production and emission processes, and developing methods for mitigating emissions from agroecosystems are essential steps in order to mitigate climate change [4].

Agricultural soils are main sources and sinks of GHG emissions, depending on their characteristics and management. Many studies have been conducted to quantify the net fluxes of CO_2_, CH_4_, and N_2_O across the soil/atmosphere interface [3], [5]–[9]. These studies provide an integrative estimate of the net production and consumption of CH_4_, CO_2_ and N_2_O in the soil, but do not provide information on the depth-distribution of CH_4_, CO_2_ and N_2_O production-consumption patterns within soil profiles. It has been suggested that subsurface processes exert a significant control on carbon (C) and nitrogen (N) dynamics and hence on CO_2_, CH_4_, and N_2_O emissions from soil [10], but few studies have elucidated the role of the subsoil so far. Understanding these processes might also provide a better insight into the possibilities and effectiveness of measures to reduce GHG emissions. For example, a temporary accumulation of GHG in the soil profile influences GHG flux patterns at the soil surface over time, and thereby may confuse empirical relationships between agricultural activities and measured GHG emissions [11]. Thus, measurements of CH_4_, CO_2_ and N_2_O concentration profiles may be helpful for increasing the understanding of the net exchanges of these gases between soil and atmosphere.

Though few studies have examined the production and consumption of CO_2_ [12], [13], CH_4_ [14] and N_2_O [15] within individual soil horizons and their transports between soil horizons so far, very few studies have made combined measurements of the dynamics of CO_2_, CH_4_ and N_2_O production and emission processes in soil profiles in agro-ecosystems, especially in China [16]. It has been well-established that N fertilizer applications increase crop growth and N_2_O emissions, and tend to decrease CH_4_ emissions into the atmosphere, but there is little information about the combined effects of N fertilizer application and irrigation on subsoil N_2_O, CO_2_ and CH_4_ production, consumption and transport.

Recently, Wang et al [16] presented bi-weekly measured CH_4_, CO_2_ and N_2_O concentration profiles down to a depth of 300 cm in a winter wheat-summer maize rotation in the North China Plain, with four N application rates (0; 200; 400 and 600 kg N ha^−1^). Here, we build on the results of that study, and present calculated subsurface fluxes of CH_4_, CO_2_ and N_2_O over a whole-year period. The purpose of this study is to evaluate the effects of seasonal cropping, N applications, irrigation, soil temperature, and soil moisture on net subsurface transport of CO_2_, N_2_O and CH_4_.

## Materials and Methods

### Site description

The study was conducted at Luancheng Agroecosystem Experimental Station (37°53′N, 114°41′E, elevation 50 m), Chinese Academy of Sciences. This area is at the piedmont of the Taihang Mountains, in the North China Plain. Mean annual precipitation is about 480 mm, 70% of which is in the period from July to September. Annual average air temperature is 12.5 °C. The dominant cropping system in the region is a winter wheat-summer maize double-cropping system (two crops harvested in a single year) without fallow between the crops.

### Field experimental design

The field experiment with a randomized complete block design was laid down in a winter wheat (*Triticum aestivum L.* Wheat variety Kenong 199)-summer maize (*Zea mays L.* Maize variety Xianyu 335) double-cropping system in 1998. It had four N fertilizer (urea) application treatments in triplicate: 0 (N0), 200 (N200), 400 (N400) and 600 (N600) kg N ha^−1^ year^−1^. Plot size was 7 m×10 m. Results of the present study refer to the period March 2007 to January 2008. Details on fertilizer application and crop management activities are presented in Table 1. Crops are flood-irrigated with pumped groundwater about five times per year, depending on rainfall distribution.

**Table 1:** Fertilization treatments (A); and timing of crop management activities (B).

(A) Treatments	Basal fertilization, applied at wheat sowing (kg·ha^−1^)			Supplementary N fertilization (kg·ha^−1^)	
	N	P_2_O_5_	K_2_O	Wheat (in April)	Maize (in July)
N0	0	65	0	0	0
N200	50	65	0	50	100
N400	100	65	0	100	200
N600	150	65	0	150	300

(B) Timing	Crop management activities				
	Winter-wheat season				Summer-maize season
October 3, 2006	Basal N fertilization and irrigation (60 mm)				
October 10, 2006	Seeding				
April 7, 2007				Supplementary N fertilization and irrigation (94 mm)	
May 19, 2007					Irrigation (60 mm)
June 1, 2007					Seeding
June 14, 2007				Harvest	
June 19, 2007					Irrigation (60 mm)
July 27, 2007					Supplementary N fertilization
July 29, 2007					Irrigation (60 mm)
October 1, 2007					Harvest
October 2, 2007	Basal N fertilization and irrigation				
October 7, 2007	Seeding				

### Soil sampling, analysis and climate data collection

The soil has a silt-loam texture in the upper 90 cm and clay-loam to clay texture at depth of 90–300 cm (Table 2). All soil samples were collected from different depths of the soil profile before the GHG measurements (on 5 December 2006); soil samples were mixed to make a specific representative soil sample for each depth; and all analyses of soil chemical properties in Table 2 were based on the standard methods for soil analyses described by Sparks [17]. Soil bulk density was determined using the cutting ring method. Soil particle size analysis was done by the Bouyoucos Hydrometer Method [18]. Soil pH was measured in a suspension of 5 g soil with 25 ml distilled water after 1 h after shaking.

**Table 2:** Soil characteristics at the experimental site in 2007.

Depth (cm)	pH (H_2_O)	Sand (%)	Silt (%)	Clay (%)	Textural class^a^	Dry bulk Density (g cm^−3^)	Total organic matter (g kg^−1^)	Total nitrogen (g N kg^−1^)	Available nitrogen (mg N kg^−1^)	Available phosphorus (mg P kg^−1^)	Available potassium (mg K kg^−1^)
0–30	8.5	25	58	17	SSL	1.47	16.7	1.40	148	4.1	79.3
30–60	7.74	22	60	18	L	1.40	10.9	0.85	72.0	2.1	54.4
60–90	7.78	31	55	14	L	1.45	7.8	0.64	70.1	0.44	36.9
90–150	7.76	15	59	26	SCL	1.57	6.5	0.38	49.8	0.17	27.8
150–200	7.74	18	47	35	GCL	1.43	5.4	0.28	38.9	0.13	21.9
200–250	7.77	15	35	50	C	1.51	4.2	0.15	30.1	0.09	13.9
250–300	7.75	12	35	53	C	1.50	3.0	0.86	25.9	0.04	7.1

a SSL: Silty sandy loam; L: Loam; SCL: Silty clay loam: GCL: Gravely clay loam; C  =  Clay.

Soil core samples were collected from different depths (0–30, 30–60, 60–90, 90–150, 150–200, 200–250, 250–300 cm) of the soil profile in the farmland described above on 5 December 2006 (before the GHG measurements), 16 June 2007 (after the winter wheat harvest) and 11 October 2007 (after the summer maize harvest), respectively. Three different sub-samples, taken from a cross-section around the soil auger (3 meter in length), were mixed to make a specific representative soil sample for each depth from each point. The soil profile samples were sealed in dark plastic bags immediately after sampling and stored at 4°C until NO_3_ extraction. Samples of soil NO_3_-N were extracted with 1 M KCl solution (1∶5 w/v) by shaking for 1 h. The extracts were then filtered and the concentrations of NO_3_-N in the soil extracts were measured colorimetrically using a UV spectrophotometer (UV-2450, Shimadzu, Japan). Each measurement was replicated three times.

Soil temperature was measured using seven CS107b soil temperature probes (Cambell Scientific Inc., Logan, UT) installed at depths of 30, 60, 90, 150, 200, 250 and 300 cm. Three-meter neutron access tubes were installed at each plot. Soil moisture at seven depths (30, 60, 90, 150, 200, 250 and 300 cm.) was measured using a neutron moisture meter when gas samples were collected. Soil temperature and water content were used to explore the relationships between calculated CO_2_, N_2_O and CH_4_ fluxes and soil water-filled pore space (WFPS) and soil temperature at various depths. Daily rainfall was recorded at a weather station on the experimental site.

### Soil gas sampling and measurements

Measurements of CO_2_, N_2_O and CH_4_ concentrations in soil started in March 2007, i.e., 9 years after the start of the field experiment, assuming that by then the CO_2_, N_2_O and CH_4_ production-consumption dynamics in the subsoil had been adjusted to the experimental treatments. Seven subsurface soil air equilibration tubes were installed at each site with sampling ports at 30, 60, 90, 150, 200, 250 and 300 cm in December 2006 (for more details, see reference 16). Soil-air samples were taken twice a week between 9:00 AM and 11:00 AM, using 100 ml plastic syringes connected to the tubes via the three-way stopcocks at the surface. The surface air was concurrently sampled at a height of 5 cm above the soil surface. The gas samples were analyzed by gas chromatography (Agilent GC-6820, Agilent Technologies Inc. Santa Clara, California, US) with separate electron capture detector (ECD at 330°C) for N_2_O determination and flame ionization detector (FID at 200°C) for CH4 and CO2 determinations.

### Calculations of gas fluxes

The basic method of our study followed that of Campbell [19]. It was assumed that the soil conditions are uniform in horizontal direction, and that the gas diffusion in soil is in one-dimensional vertical flow, that fundamentally follows Fick’s law [20], [21]:
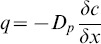
(1)


Where *q* is the gas flux density (g gas m^−2^ soil s^−1^), *D_p_* is the soil-gas diffusivity (m^3^ soil air m^−1^ soil s^−1^), 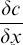 is the concentration gradient between two soil layers (g gas m^−3^ soil air m^−1^ soil).

*D_p_* was derived from the following equation [22], [23]: 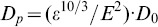
(2)


Where, *D_0_* is the gas diffusivity (m^2^ air s^−1^). We estimated the diffusion coefficient *D_0_* of CH_4_, CO_2_ and N_2_O at 298 K and 1 kPa at 1.79×10^−5^, 1.32×10^−5^, 1.29×10^−5^ m^2^ s^−1^, respectively, by using a semiempirical equation by Gilliland et al [24]. Parameter *ε* is the soil air filled porosity (m^3^ air m^−3^ soil), and *E* is the soil porosity (m^3^ voids m^−3^ soil).

The Millington-Quirk model was used to compute *ε* and *E* [25]:
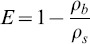
(3)

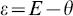
(4)


Where *ρ_b_* is the dry bulk density (g m^−3^) at each soil depth (Table 2), *ρ_s_* is the average bulk density of surface soil (2.65 g m^−3^); *θ* is the volumetric soil water content which was measured using a neutron moisture meter at each depth.

### Calculations of annual cumulative gas fluxes

The annual cumulative emissions were obtained by multiplying the average daily flux from two consecutive measurements within a week by the number of days between the measurements, and then summing the fluxes of these periods to an accumulative flux for the whole year [26]:
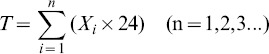
(5)


Where: *T* (kg ha^−1^), *X_i_* (kg ha^−1^ h^−1^) and *i* are the accumulative GHG emission, the average daily GHG emission rate, and the number of days, respectively.

### Data analyses

All data were subjected to statistical analysis (SPSS 13.0). Differences between treatments were analyzed using ANOVA, followed by LSD at the 0.05 probability level. Regression analysis was used to identify relationships between CH_4_, CO_2_ and N_2_O fluxes and the climatic variables.

## Results

### Concentrations of CH_4_, CO_2_ and N_2_O

**Figure 1:**
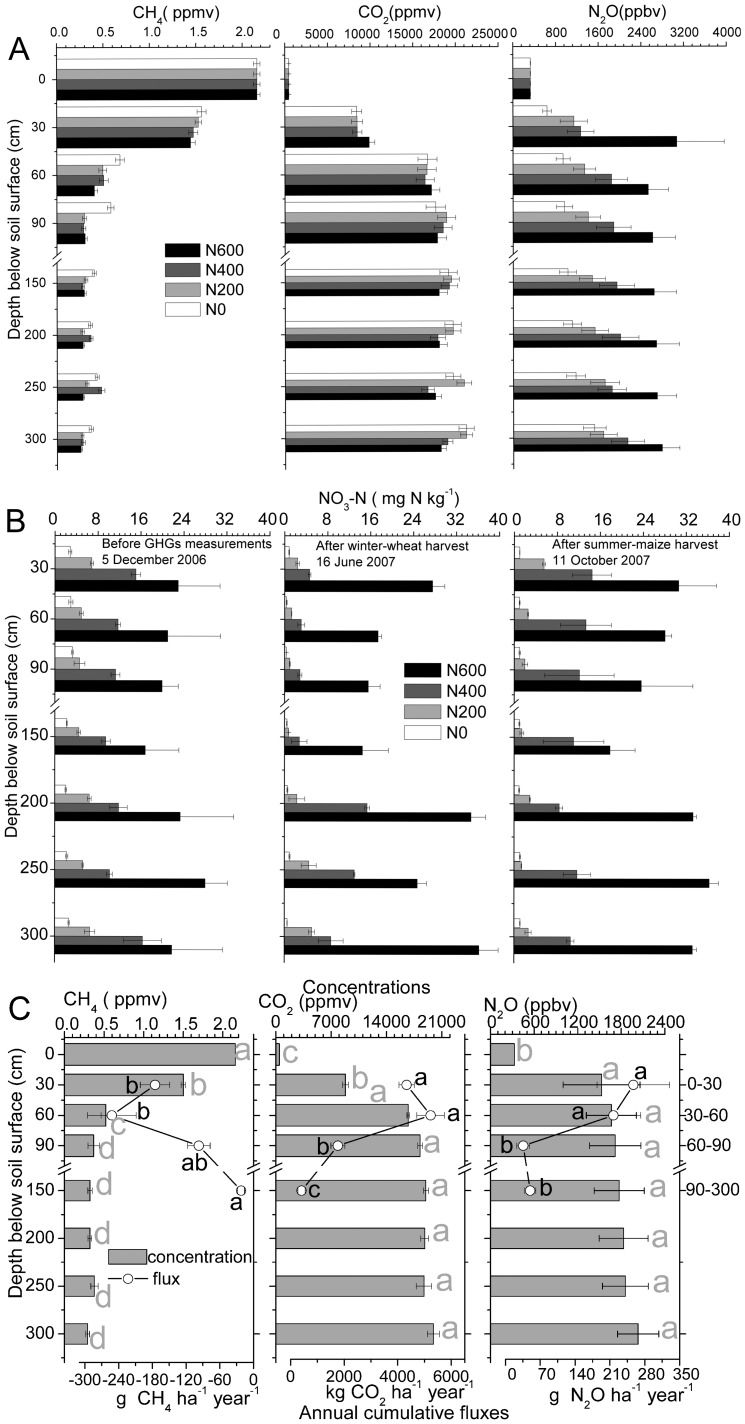
CH_4_, CO_2_ and N_2_O concentrations (mean ± standard deviations, n = 3) in soil air at various soil depths in a winter wheat–summer maize double cropping rotation receiving 0, 200, 400 and 600 kg of N ha^−1^ year^−1^, in 2007–2008 (A); NO_3_-N contents (mean ± standard deviations, n = 3) at various soil depths as function of N fertilizer application rate, in 2007–2008 (B); Profiles of concentration and annual cumulative flux of CH_4_, CO_2_ and N_2_O, in 2007–2008 (mean ± standard deviations, n = 4). Same letters next to the bars indicated no significant differences between slope positions (P<0.05). (C). Note the differences in X-axes.

Mean concentrations and its standard deviations of CH_4_, CO_2_ and N_2_O at each depth are shown in Figure 1 A, as function of N application rates. Mean CH_4_ concentration decreased with soil depth. Ambient air CH_4_ concentration in the area was about 2.2 ppmv. At a depth of 30 cm, CH_4_ concentration ranged between 1.4 and 1.6 ppmv and at depth of 60 to 300 cm between 0.3 and 0.6 ppmv. There were no clear effects of N fertilizer application on the mean CH_4_ concentration (Figure 1 A). Mean CH_4_ concentrations decreased significantly at soil depths of 0, 30, 60 and 90 cm (P<0.05); changes in mean concentration below a depth of 90 cm were not significant (Figure 1 C).

Mean CO_2_ concentration increased with soil depth. At a depth of 30 cm, CO_2_ concentration ranged between 8400 and 9900 ppmv and at depth of 60 to 300 cm between 16000 and 21000 ppmv. Mean CO_2_ concentrations increased significantly at soil depths of 0, 30 and 60 cm (P<0.05); changes in mean concentration below a depth of 60 cm were not significant (Figure 1 C). There were no clear effects of N fertilizer application on the mean CO_2_ concentrations.

Concentrations of N_2_O were strongly influenced by agricultural management activities such as N application and irrigation. Fertilizer N applications increased the mean N_2_O concentrations. Mean N_2_O concentrations at depth of 30 to 300 cm ranged from 600 to 1500, 1100 to 1700, 1600 to 2100 and 2500 to 3000 ppbv for the N0, N200, N400 and N600 treatments, respectively (Figure 1 A). Mean N_2_O concentrations increased significantly from soil surface to a depth of 30 cm (P<0.05), but changes in mean concentration below a depth of 30 cm were not significant (Figure 1 C). Fertilizer N application increased soil NO_3_-N content; differences in mean N_2_O concentrations were correlated with differences in mean NO_3_-N contents in the four fertilizer N treatments (Figure 1 B).

### Fluxes of CH_4_ in soil

**Figure 2:**
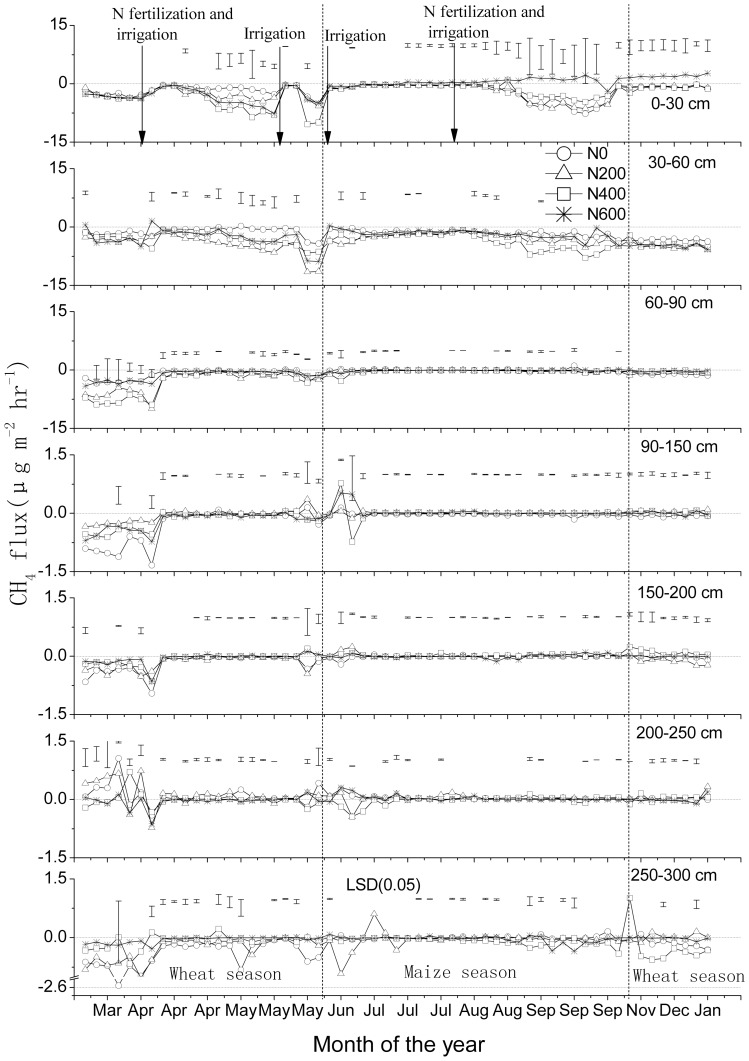
CH_4_ flux rates (means ± standard deviations, n = 3) at various soil depths in a winter wheat-summer maize double cropping rotation receiving 0, 200, 400 and 600 kg of N ha^−1^ year^−1^, in 2007–2008. Vertical dashed lines indicate a change in crop. Bars in figures indicate 1 standard deviation (n = 3). Note the differences in Y-axes.

Diffusive fluxes between soil layers and between soil and atmosphere were calculated from the concentration gradients, using equation 1. There was a net influx of atmospheric CH_4_ into the top 0–60 cm (Figure 2), suggesting consumption of CH_4_ by methanotropic bacteria. Interestingly, the calculated fluxes into the soil were rather similar for the 0–30 and 30–60 cm soil layers, suggesting similar CH_4_ uptake rates. Uptake of CH_4_ apparently also occurred in the layers 60–90, 90–150 and 150–200 cm during the first one or two months of the measurement period (Figure 2). However, we cannot exclude the possibility that this apparent uptake of CH_4_ in the subsoil during the first two months is an artifact related to the installation of the samplers when atmospheric CH_4_ may have diffused into the subsoil. Fluxes between soil layers were negligible small during most of the maize growing season (Figure 2).

Annual cumulative fluxes of CH_4_ for all soil layers and N fertilizer treatments are shown in Table 3 A. Evidently, the influx of atmospheric CH_4_ decreased with soil depth. During the study period, mean calculated uptake was about 176 g CH_4_ per ha by the top 30 cm, 252 g CH_4_ per ha by the soil layer 30–60 cm, 98 g CH_4_ per ha by the layer 60–90 cm and 22 g CH_4_ per ha below a depth of 90 cm; mean calculated uptakes in the layers 0–30 and 30–60 cm were both significantly higher than that in the layer 90–300 cm (P<0.05) (Figure 1 C). Annual cumulative CH_4_ uptake in the layer 0–90 cm (526 g CH_4_ per ha per year) contributed about 96% to that in the layer 0–300 cm (547 g CH_4_ per ha per year). Annual cumulative uptake in 0–30 cm layer is relatively low compared to literature data [4]–[6], [27].

**Table 3:** Annual cumulative emissions of CH_4_ and N_2_O (in g ha^−1^ yr^−1^) and of CO_2_ (in kg ha^−1^ yr^−1^) between soil layers.

(A) CH_4_								
Treatments	0–30 cm	30–60 cm	60–90 cm	90–150 cm	150–200 cm	200–250cm	250–300 cm	0–300cm
N0	−167 (12) b	−138 (13) a	−63 (2) a	−15 (0.5) c	−9 (0.1) d	5 (0.04) a	−21 (0.3) d	−408
N200	−201 (9) bc	−322 (10) c	−123 (3) b	−2 (0.07) a	−7 (0.5) c	5 (0.08) a	−12 (0.6) c	−662
N400	−228 (3) c	−318 (8) c	−140 (10) b	−6 (0.5) b	−1 (0.1) a	−1 (0.06) b	−10 (0.4) b	−704
N600	−106 (15) a	−231 (5) b	−64 (7) a	−6 (0.5) b	−2 (0.1) b	−1 (0.05) b	−4 (0.1) a	−414

(B) CO_2_								
Treatments	0–30 cm	30–60 cm	60–90 cm	90–150 cm	150–200 cm	200–250cm	250–300 cm	0–300cm
N0	4,986 (401) a	6,060 (394) a	1,730 (121) b	163 (9) a	92 (1) a	12 (0.1) b	294 (15) a	13,337
N200	3,819 (652) a	6,019 (424) b	2,194 (177) a	194 (19) a	19 (0.5) c	263 (8) a	198 (4) c	12,706
N400	4,640 (225) a	4,955 (174) c	2,100 (112) ab	152 (15) a	−48 (1) d	−1 (0.01) c	251 (12) b	12,049
N600	3,880 (383) a	3,874 (147) c	1,049 (120) c	11 (2) b	33 (0.6) b	−79 (0.5) d	116 (4) d	8,884

(C) N_2_O								
Treatments	0–30 cm	30–60 cm	60–90 cm	90–150 cm	150–200 cm	200–250cm	250–300 cm	0–300cm
N0	93 (7) c	90 (2) d	23 (3) b	6 (0.3) a	9 (0.3) b	4 (0.4) d	62 (1) a	287
N200	226 (23) b	199 (7) c	24 (1) b	−1 (0.02) b	12 (0.6) a	51 (2) a	−20 (2) d	491
N400	263 (27) b	358 (5) a	23 (2) b	−6 (0.2) d	−2 (0.2) c	20 (1) b	35 (0.5) b	691
N600	447 (1) a	222 (2) b	74 (4) a	−3 (0.1) c	−2 (0.1) c	11 (0.7) c	22 (2) c	771

Values (means with SE in the brackets) followed by the same letter are not significantly different within columns (one-way ANOVA with LSD; P<0.05)

### Fluxes of CO_2_ in soil

**Figure 3:**
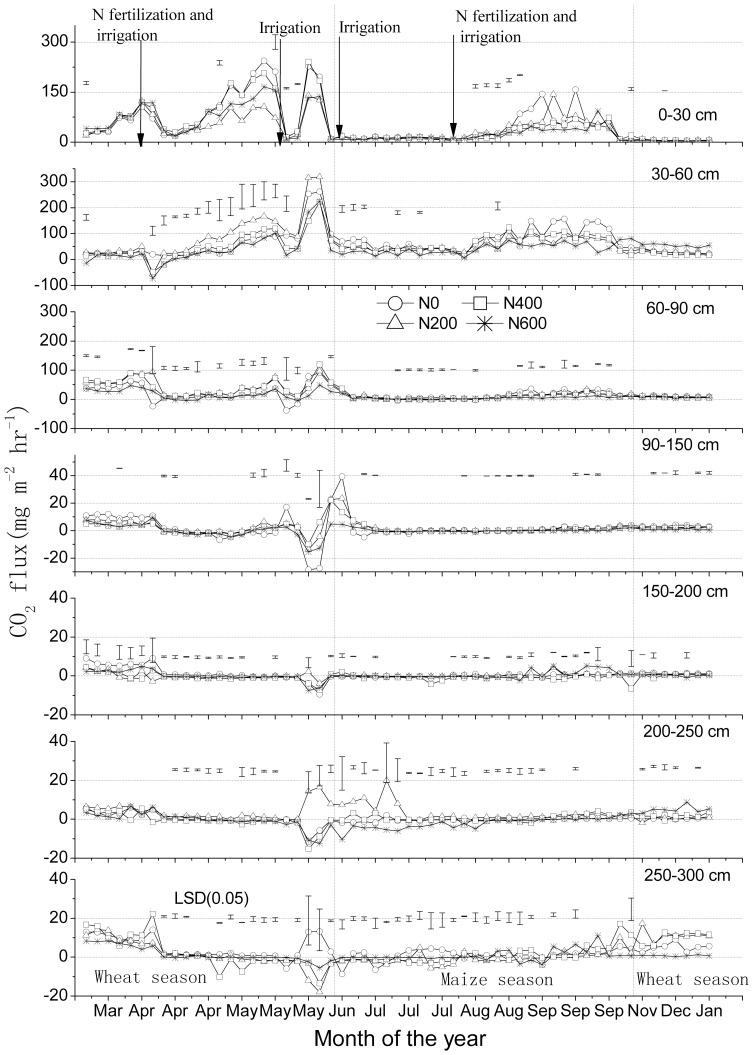
CO_2_ flux rates (means ± standard deviations, n = 3) at various soil depths in a winter wheat-summer maize double cropping rotation receiving 0, 200, 400 and 600 kg of N ha^−1^ year^−1^, in 2007–2008. Vertical dashed lines indicate a change in crop. Bars in figures indicate 1 standard deviation (n = 3). Note the differences in Y-axes.

There was a large efflux of CO_2_ from the top 30 cm of soil to the atmosphere from March till June, i.e., during the second half of the wheat growing season, and from August till October, i.e., during the second half of the maize growing season (Figure 3). The same holds for the upward flux from the layer 30–60 to the layer 0–30 cm. These patterns were related to the crop growing seasons of wheat and maize, and to the changes in water filled pore space (WFPS) and soil temperature. There were no clear relationships between N treatments and CO_2_ fluxes. Treatment N200 had the smallest flux from the layer 0–30 cm to the atmosphere, but the largest from 30–60 cm to 0–30 cm from April to May. Upward fluxes from the layers 60–90 cm and especially below this layer were much smaller. There were small but significant changes in fluxes in the subsoil at the transition of the winter-wheat growing season to the summer-maize growing season (Figure 3).

Surprisingly, annual cumulative fluxes of CO_2_ tended to decrease with increasing N fertilizer application rates (Table 3 B). Moreover, cumulative upward fluxes were somewhat larger from the layer 30–60 cm (mean 5,227; range 3,800–6,000 kg CO_2_ per ha) than from the layer 0–30 cm to the atmosphere (mean 4,331; range 3,800–5,000 kg CO_2_ per ha) (Figure 1 C; Table 3 B). This suggests that a relatively large portion of total respiration in soil took place in the layer 30–60 cm. However, we can not exclude the possibility that the calculated CO_2_ efflux from the top layer is underestimated, because the concentration gradient in the upper 0–30 cm soil layer was averaged, and soil diffusivity may be higher in the top few cm than the bulk of the top 30 cm of soil [28]. Annual cumulative CO_2_ flux in the layer 0–90 cm (11,327 kg CO_2_ per ha per year) contributed about 97% to that in the layer 0–300 cm (11,744 kg CO_2_ per ha per year). Mean calculated fluxes in the layers 0–30, 30–60 and 60–90 cm were all significantly higher than that in the layer 90–300 cm (P<0.05); mean annual cumulative fluxes from the soil below 90 cm were very small and in upwards direction (Figure1 C; Table 3 B).

### Fluxes of N_2_O in soil

**Figure 4:**
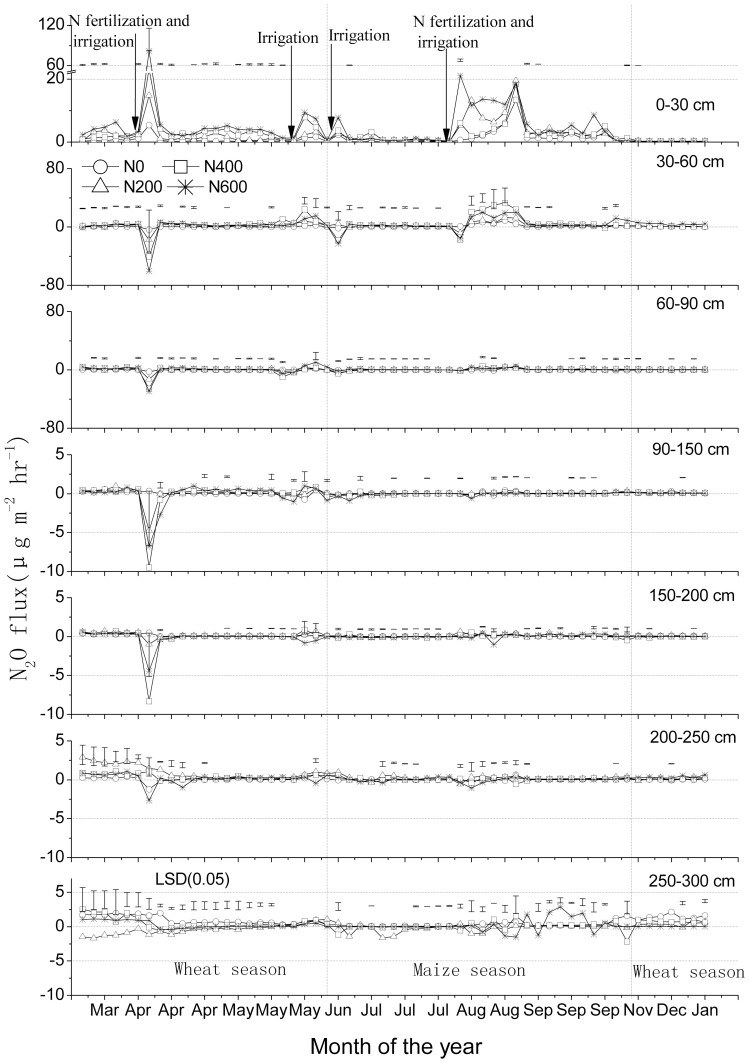
N_2_O flux rates (means ± standard deviations, n = 3) at various soil depths in a winter wheat-summer maize double cropping rotation receiving 0, 200, 400 and 600 kg of N ha^−1^ year^−1^, in 2007–2008. Vertical dashed lines indicate a change in crop. Bars in figures indicate 1 standard deviation (n = 3). Note the differences in Y-axes.

**Figure 5:**
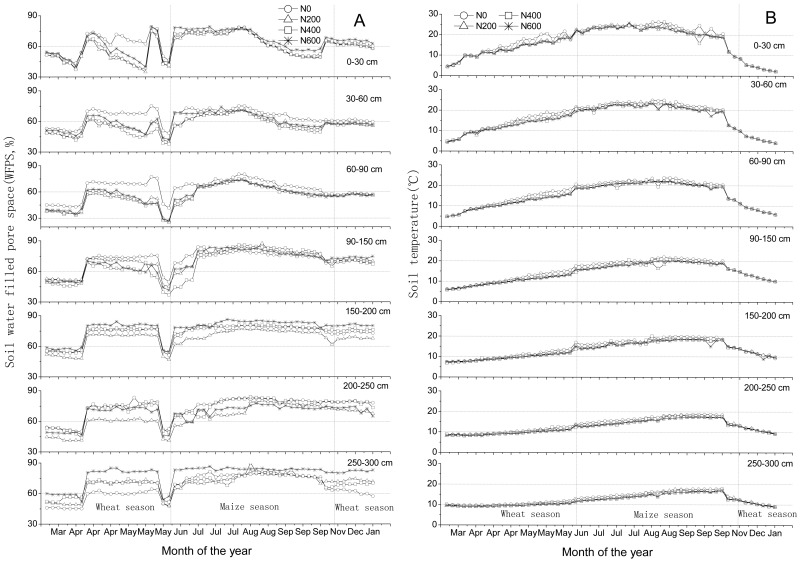
Water-filled pore space (WFPS) at various soil depths in a winter wheat-summer maize double cropping rotation receiving 0, 200, 400 and 600 kg of N ha^−1^ year^−1^, in 2007–2008. Bars in figures indicate 1 standard deviation (n = 3). (A); Soil temperatures at various soil depths in winter wheat-summer maize double cropping rotation receiving 0, 200, 400 and 600 kg of N ha^−1^ year^−1^, in 2007–2008.(B)

Fertilizer application, irrigation and precipitation events triggered an efflux of N_2_O from the topsoil to the atmosphere (Figure 4). The peak efflux, associated with the supplemental N fertilizer application and flooding in early April (wheat growing season), was accompanied with significant downward directed fluxes below the topsoil layer (0–30 cm). There was another relatively large efflux of N_2_O into the atmosphere during the relatively moist and warm August summer month (maize growing season) (Figures 4 and 5), but this peak was not accompanied with significant downward directed fluxes below the topsoil layer. In the subsoil, fluxes were relatively small and directions variable (Figure 4). Essentially all seasonal fluctuations of N_2_O flux rates in the subsoil (60–200 cm) seem to be related to fertilizer application, irrigation and rainfall events and changes in WFPS; therefore, there was no clear evidence of N_2_O production in the subsoil after excluding these influence of interfering factors [16] **.**

Annual cumulative fluxes of N_2_O increased with increasing N fertilizer application rates; calculated total emissions at the soil surface were 93, 226, 263 and 447 g N_2_O per ha for the N0, N200, N400 and N600 treatments, respectively; net upward fluxes from the 30–60 cm layer were almost as large (90, 199, 358 and 222 g N_2_O per ha for the N0, N200, N400 and N600 treatments, respectively) as the fluxes from the 0–30 cm layer to the atmosphere (Table 3 C). Mean calculated fluxes in the layers 0–30 and 30–60 cm were both significantly higher than those in the layers 60–90 and 90–300 cm (P<0.05) (Figure 1 C); mean annual cumulative fluxes from the soil below 90 cm were small but mostly in upwards direction, suggesting that the subsoil was a small source of N_2_O, and/or that accumulated N_2_O from the previous season contributed to the net upward directed fluxes.

### Relations between WFPS and temperature and CH_4_, CO_2_ and N_2_O fluxes

Linear regression relationships between WFPS and CH_4_ fluxes (positive) and between WFPS and CO_2_ fluxes (negative) were statistically significant (p<0.05) for almost all soil layers (Table 4). Uptake of CH_4_ by the soil was relatively high when WFPS was relatively low, probably because the diffusion rate of CH_4_ into the soil was high when soil was dry, and vice versa [6]. Similarly, the upward transport of CO_2_ was low when WFPS was high, and vice versa. This indicates that soil moisture exerted a dominant control on CH_4_ and CO_2_ fluxes. The linear relationship between WFPS and N_2_O flux was also significant for the soil layers 200–250 and 250–300 cm (p<0.05), but not for the other layers. Significant downward directed fluxes below the topsoil were only observed down to a deep of 200 cm (Figure 4); note that N_2_O fluxes were very low in 200–300 cm soil layer. Apparently, in 200–300 cm deep soil profile, soil moisture exerted a dominant control on nitrification and denitrification processes. But in 0–200 cm soil layer, WFPS was not the dominant controlling factor for the diffusive N_2_O flux, likely the combination of WFPS, ammonia, nitrate and metabolizable carbon, because these factors commonly control nitrification and denitrification processes.

**Table 4:** Linear regressions for the relationship between climatic variables and GHG fluxes.

Climatic variable	Soil depth (cm)	CH_4_ (μg m^−2^ hr^−1^)	CO_2_(mg m^−2^ hr^−1^)	N_2_O (μg m^−2^ hr^−1^)
Soil water filled pore space	0–30	0.853**	−0.775**	−0.149
	30–60	0.645**	−0.372**	0.084
	60–90	0.787**	−0.852**	0.067
	90–150	0.637**	−0.447**	0.093
	150–200	0.771**	−0.268*	0.084
	200–250	−0.146	−0.289*	−0.692**
	250–300	0.763**	−0.532**	−0.579**
Soil temperature	0–30	−0.001	0.042	0.104
	30–60	0.270*	0.356**	0.211
	60–90	0.635**	−0.348**	0.093
	90–150	0.620**	−0.225	0.062
	150–200	0.575**	−0.266*	−0.075
	200–250	−0.122	−0.299*	−0.493**
	250–300	0.473**	−0.323*	−0.263*

Pearson's correlation coefficient, 2-tailed tests of significance.

^**^Significant correlation at a <0.01.

^*^Significant correlation at a <0.05.

Relationships between soil temperature and CH_4_, CO_2_ and N_2_O fluxes showed relatively large scatter (Table 4). Evidently, high temperatures are associated with the summer season, which is relatively moist (Figure 5). The significant relationships (p<0.05) between soil temperature and CH_4_ fluxes at depth of 60–300 cm may be the result in part of the covariance between WFPS and soil temperature. Fluxes of N_2_O in soil were not significantly related to soil temperature (Table 4).

## Discussion

Fluxes of CH_4_, CO_2_ and N_2_O at the interface of soil and atmosphere are the net result of production, consumption and transport in the soil [11]. In this study, we inferred fluxes in the soil profile from changes in concentrations with depth and over time, so as to identify soil horizons of CH_4_, CO_2_ and N_2_O production and consumption, and thereby to increase the understanding of the dynamics of the net fluxes at the interface of soil and atmosphere. The study is unique in the sense that the inference of subsurface fluxes of CH_4_, CO_2_ and N_2_O in 300 cm deep soil profiles at high temporal resolution over a full year has not been reported before in such comprehensive manner.

Though the C and N cycles are intimately linked in the biosphere, there were significant differences in the dynamics of CH_4_, CO_2_ and N_2_O production, consumption and transport in the studied soil. The concentration profiles have distinct characteristics (Figure 1 A); the seasonal dynamics were much larger in the topsoil than subsoil. Moreover, the seasonal dynamics in inferred fluxes occurred during distinct periods (Figures 2, 3 and 4), and these were related to changes in WFPS (Figure 5 A), following rainfall and irrigation events. Fertilizer N application affected N_2_O fluxes greatly, but not those of CH_4_ and CO_2_. The soil under the winter wheat-summer maize double cropping system was a net sink of atmospheric CH_4_ and a net source of N_2_O. It was also a large source of CO_2_ but it is unknown whether the efflux compensated the influx of C into the soil via plant growth, as the latter influx was not measured.

The inferred fluxes at the soil-atmosphere interface (Figure 1 C; Table 3) were relatively small compared to those observed in other studies [5], [6], [29]. Our estimated soil surface fluxes are very likely underestimates because the depth resolution of the gas samplers in the top soil was too low to capture the curvature of the concentration profile properly. Hence, our study may have underestimated the dynamics of the CH_4_, CO_2_ and N_2_O fluxes in the top soil. The depth resolution of the sampling below 90 cm appeared to be adequate. Below, we discuss the dynamics of the CH_4_, CO_2_ and N_2_O fluxes in the soil profile in more detail.

### CH_4_ flux

Application of fertilizer N has been shown to inhibit CH_4_ oxidation in soil [30], [31], and several studies noted that non amended soils act as sink of CH_4_ [32]–[34]. In our study, seasonal mean emission rates and annual cumulative fluxes of CH_4_ for all soil layers and N fertilizer treatments were consistently directed downward (Figures 1 C and 2; Table 3 A). Though statistical significant differences in cumulative CH_4_ fluxes between fertilizer N treatments were observed (Table 3 A), there was no clear trend that an increase in total N application decreased CH_4_ uptake by soil. Inferred uptake was higher in the N200 and N400 treatments than in the N0 and N600 treatments at depth of 30 to 90 cm.

The magnitude of methane uptake by soils is largely controlled by diffusion of atmospheric methane into the soil [35], which in turn is strongly influenced by soil moisture [28]. The rate of diffusion of CH_4_ in soil was high when WFPS was low. Our results showed a significant negative linear correlation between CH_4_ uptake rate and WFPS for almost all layers; and the highest CH_4_ uptake rates took place when WFPS was under 70% (Figures 2 and 5 A; Table 4). This is in agreement with the studies by Guo et al, Wu et al and Wang et al [4], [6], [16]. Inferred downward CH_4_ fluxes decreased with depth (Figures 1 C and 2; Table 3 A). It has been reported that methanotrophic activity is most pronounced in the top soil [4], [27], but our study suggests that significant uptake took place up to depths of 60 to 90 cm; below 90 cm, inferred fluxes of CH_4_ were negligibly small (Figure 1 C).

### CO_2_ flux

Application of 200 kg fertilizer N per ha per year and more roughly doubled grain yields relative to the control treatment [16], but did not have statistical significant effects on the CO_2_ efflux from the soil and the diffusive flux in the soil profile (Figure 3; Table 3 B). Apparently, fertilizer N application affected predominantly aboveground biomass production, and not so much underground biomass production and respiration. Yet, we may have missed some of the topsoil dynamics, also because the incorporation of the stubbles by ploughing was in the top 15 cm of soil only. A relatively large portion of total respiration in soil took place in the layer 30–60 cm; and the total respiration in the layer was significantly higher than those in the layers 60–90 and 90–300 cm (P<0.05) (Figure 1 C).

When soil WFPS ranged between 40 and 70% and soil temperature was >10 °C (Figure 5), highest CO_2_ fluxes took place at depth of 0–60 cm during the second half of the growing seasons of wheat and maize, i.e., from mid-April to mid-May and from mid-August to mid-September (Figures 3 and 5 A). These elevated CO_2_ emissions are attributed to root respiration and to enhanced mineralization of soil organic matter by increased microbial activity [36], but also to changes in the stability and formation of soil aggregates and in the microbial community structure [37]. Sufficient soil moisture is needed to allow and support substrate diffusion to the sites of microbial activity. However, if soil moisture values exceed certain thresholds (which do depend on soil properties such as porosity, bulk density and SOC content) microbial soil respiration can get O_2_ limited due to diffusion constrains [6]. In a saturated soil, air is pushed out of soil pore spaces and root respiration further depletes O_2_ in the soil air [38], [39]. In our study, a significant negative linear correlation was found between CO_2_ flux rate and WFPS (40–70%) in all layers (Table 4); and low CO_2_ fluxes took place when WFPS exceeded 70%, especially after irrigation or heavy rainfall events, i.e., from June to August (Figures 3 and 5 A). This assertion is also supported by results presented by Davidson et al, Jassal et al and Fang et al [38]–[40].

Generally, the CO_2_ evolution from soil is directly correlated with soil temperature, though within a certain temperature range [41], [42], and depending on the presence of active roots [43]. In our study, the relationship of soil temperature and CO_2_ fluxes was variable, likely because of the dominant effect of WFPS (Table 4).

### N_2_O flux

Nitrogen application and irrigation/rainfall are main triggers for increased N_2_O concentrations in a soil profile and for increased emissions [15], [16]. The top soil was the source of N_2_O production. The combined urea applications and irrigations in early April and by the end of July 2007 strongly increased NO_3_^−^ (Figure 1 B), NH_4_^+^ (data not shown) contents and WFPS (Figure 5 A) in soil, and induced large upward directed fluxes in the upper 0–30 cm soil layer; interestingly, relatively large downward directed fluxes in the subsoil only took place in April (up to the depths of 60 and 90 cm) (Figure 4), we cannot exclude the possibility that the apparent downward directed peaks during the first two months probably related to the soil structure disturbance that resulted from the installation of the samplers in December 2006.

In soil, N_2_O is mainly produced by nitrification and denitrification processes. The most important factors controlling these processes are NH_4_^+^ and NO_3_^−^ contents, O_2_ partial pressure, and available carbon to fuel heterotrophic denitrification [44], [45]. The rapid increases of WFPS (Figure 5 A), NO_3_-N content (Figure 1 B) and N_2_O productions in the subsoil (Figures 1 A and 4) would suggest that convective transport contributed to the downward transport of water and solutes (especially in the maize growing season), which is in line with other observations [2], [46]. For instance, significant upward directed fluxes of N_2_O took place during the relatively moist and warm August (maize growing season) than in the preceding wheat season in 30–60 cm soil layer (Figure 4). Several studies have demonstrated that higher values of soil moisture and temperature result in higher N_2_O fluxes [44]–[46] Also, Li et al found while carrying out a three-year field experiment at the same study sites that significant NO_3_- leaching events occur predominantly during August to October (maize growing season) [47]. Zhu et al found while carrying out a four-year field experiment in a hillslope cropland that soil NO_3_- concentrations in the subsurface soil (15–30 cm) were higher than in the topsoil (0–15 cm) during most of the maize season, indicating a rapid and effective transport of NO_3_- to the subsurface soil following over irrigation or rainfall events [48]. Our results indicate that NO_3_-N contents in soil layers after the maize harvest were higher than after the wheat harvest (Figure 1 B); but due to missing measurements, we can not fully rule out that high NH_4_^+^ content [47] may have contributed to relatively high N_2_O emissions in the warm and wet maize season. Although N_2_O concentration increased with soil depth, changes in inferred N_2_O flux below a depth of 60 cm were relatively small (Figures 1 A and 4). It may be related to the variation of the vertical N_2_O concentration gradient; changes in mean N_2_O concentration below a depth of 30 cm were not significant (Figure 1 C).

It has been frequently observed that high rates of N_2_O emissions take place when WFPS ranges between 30 and 70% [49]. According to Zou et al the N_2_O production in dry land soil of Northern China is mostly driven by nitrification [50]. Wang et al suggests that nitrification is likely a main source of the N_2_O production in the soil profile when WFPS varied between 45 and 70% at the study site [16]. N_2_ starts being emitted through denitrification at a WFPS of 70%, and is the main N gas emitted when WFPS exceeds 75% [51]. This may explain the relatively high inferred N_2_O flux from late April to mid-May (WFPS, 40–70%) and the very low flux from late June to late July (before applying nitrogen) (WFPS, >70%) in the layer 0–30 cm (Figures 4 and 5 A).

The accumulated N_2_O fluxes were significantly related to N application rate. This was most apparent in the top 30 to 90 cm of soil (Table 3 C). Annual cumulative N_2_O flux in the layer 0–90 cm (511 g N_2_O per ha per year) contributed about 90% to that in the layer 0–300 cm (560 g N_ 2_O per ha per year). The 90 cm thick cinnamon top soil overlays the so-called Shajiang layer (90–140 cm) with silty clay loam texture [52]. The Shajiang layer has no crop roots, contains many iron-manganese nodules and has high bulk density (Table 2). This compacted subsoil may explain that fertilizer application and irrigation mainly affected N_2_O fluxes down to 90 cm (Figure 1 C; Table 3 C). In this study, calculated total emissions in the layer 0–90 cm were 206, 449, 644 and 743 g N_2_O per ha for the N0, N200, N400 and N600 treatments, respectively; these fluxes translate into fertilizer-derived emissions of 0.14, 0.10 and 0.07% for the N200, N400 and N600 treatments, respectively. The fertilizer induced emission factors (0.07–0.14%) were lower than the 0.30–0.39% measured by Ding et al [53] over the maize-wheat rotation year in a long-term mineral nitrogen addition field experiments (150–300 kg N ha^−1^ year^−1^, over 20-years) in the North China Plain. A reason for the lower fertilizer induced emission factor is probably related to the likely underestimates of soil surface N_2_O fluxes, because the concentration gradient in the upper 0–30 cm soil layer was averaged, and soil diffusivity may be higher in the top few cm than the bulk of the top 30 cm of soil [28]. Furthermore, due to missing measurements we can not fully rule out the indirect N_2_O emissions from leaching and atmospheric deposition [47], [53].

## Conclusions

Our study is one of few that inferred CH_4_, CO_2_ and N_2_O transport between soil layers from changes in CH_4_, CO_2_ and N_2_O concentrations in the upper 300 cm of soil, measured at (bi)-weekly time intervals for one year in a winter wheat-summer maize double crop rotation. The top 30 to 60 cm of soil was a sink of atmospheric CH_4_, and a source of both CO_2_ and N_2_O. There was little or no evidence that the subsoil (>90 cm) acted as a sink or source of GHG; rather it acted as “reservoir”.

Nitrogen fertilizer application increased N_2_O fluxes but did not affect CH_4_ and CO_2_ fluxes. The fertilizer-derived N_2_O flux was small, likely because our sampling design may have missed N_2_O production in the top 15 cm of soil. This holds as well for the CH_4_ consumption by soil and the CO_2_ emissions from soil; both are likely underestimated. Soil moisture (WFPS) was found to play an important regulating role for CH_4_, CO_2_ and N_2_O fluxes in soil and between soil and atmosphere. Both CH_4_ consumption and CO_2_ fluxes in and from soil all tended to decrease with increasing WFPS.

More than 90% of the annual cumulative GHG fluxes originated at depths shallower than 90 cm. Mostly because the productive soil of our study site in the North China Plain had two distinct layers (0–90 and >90 cm), with different texture and bulk density. These differences showed up in characteristic differences in GHG concentration profiles and fluxes.
